# Supplementary material for: Application of Graph Models to the Identification of Transcriptomic Oncometabolic Pathways in Human Hepatocellular Carcinoma
Source: Biomolecules. 2024 Jun 3;14(6):653. doi: 10.3390/biom14060653 (PMC11201933; doi:10.3390/biom14060653)
Supplement: Supplementary file 1 [file biomolecules-14-00653-s001.zip › Barace_Biomolecules_SupplTable1.pdf]

Table S1. Clinical variables of TCGA-LIHC and ICGC-LIRI-JP cohorts.

Clinical variable	TCGA-LIHC n=365	ICGC-LIRI-JP n=200	Test[1]
**Tumor type**			
Hepatocellular carcinoma	361 (98.90%)	200 (100.00%)	Fisher test
Hepatocellular carcinoma – clear cell type	4 (1.10%)	0 (0.00%)	p=0.303
**Gender**			
Male	248 (67.95%)	150 (75.00%)	Fisher test
Female	117 (32.05%)	50 (25.00%)	p=0.083
**Age**			
< 65 years	215 (58.90%)	72 (36.00%)	Fisher test
≥ 65 years	149 (40.82%)	128 (64.00%)	p<0.001
NA[2]	1 (0.30%)	0 (0.00%)	
**Race**			
Caucasic – White	164 (44.93%)	0 (0.00%)	
Asian	158 (43.29%)	200 (100.00%)	X^2 test
Black or african american	17 (4.66%)	0 (0.00%)	p<0.001
Hispanic or latino	17 (4.66%)	0 (0.00%)	**NA out of test*
American indian or Alaska native	2 (0.54%)	0 (0.00%)	
Not reported	7 (1.92%)	0 (0.00%)	
**Hepatitis infection**			
HBV	57 (15.62%)	53 (26.50%)	X^2 test
HCV	18 (4.93%)	113 (56.50%)	p<0.001
HBV and HCV	81 (22.19%)	4 (2.00%)	**NA out of test*
None	209 (57.26%)	30 (15.00%)	
**Alcohol consumption**			
No or social drinker	58 (15.89%)	108 (54,00%)	Fisher test
Yes	109 (29.86%)	83 (41.50%)	p<0.001
NA	198 (54.25%)	9 (4.50%)	**NA out of test*
**Edmondson stage**			
G1	53 (14.52%)	20 (10.00%)	
G2	173 (47.40%)	115 (57.50%)	X^2 test
G3	122 (33.42%)	21 (10.50%)	p<0.001
G4	12 (3.29%)	1 (0.5%)	**NA out of test*
Undetermined	0 (0.00%)	42 (21.00%)	
NA	5 (1.37%)	1 (0.5%)	
**TNM stage**			
T1	179 (49.04%)	19 (9.50%)	
T2	91 (24.93%)	112 (56.00%)	X^2 test
T3	79 (21.64%)	20 (10.00%)	p<0.001
T4	13 (3.56%)	1 (0.5%)	**NA out of test*
Undetermined	0 (0.00%)	47 (23.50%)	
NA	3 (0.83%)	1 (0.5%)	
**Hepatic fibrosis**			
No fibrosis	73 (20.00%)	10 (5.00%)	
Mild fibrosis	31 (8.49%)	27 (13.50%)	X^2 test
Moderate fibrosis	30 (8.22%)	40 (20.00%)	p<0.001
Severe fibrosis	81 (22.19%)	123 (61.50%)	**NA out of test*
NA	150 (41.10%)	0 (0.00%)	
**TP53 mutation**			
No	256 (70.14%)	124 (62.00%)	Fisher test
Yes	109 (29.86%)	76 (38.00%)	p=0.06

**CTNNB1 mutation**			
No	270 (73.97%)	120 (60.00%)	Fisher test
Yes	95 (26.03%)	80 (40.00%)	p<0.001
**TERT mutation**			
No	281 (76.99%)	175 (87.50%)	Fisher test
Yes	84 (23.01%)	25 (12.50%)	p=0.002

[1] Chi Square Test, [2] Not Available Data
