# Supplementary material for: Application of Graph Models to the Identification of Transcriptomic Oncometabolic Pathways in Human Hepatocellular Carcinoma
Source: Biomolecules. 2024 Jun 3;14(6):653. doi: 10.3390/biom14060653 (PMC11201933; doi:10.3390/biom14060653)
Supplement: Supplementary file 1 [file biomolecules-14-00653-s001.zip › Barace_Biomolecules_SupplTable2.pdf]

Table S2. Characteristics of HCC cell lines from DepMap Portal.

ModelID	CellLineName	DepmapModelType	Age	AgeCategory	Sex	PatientRace	PrimaryOrMetastasis	CCLEName	COSMIC ID
ACH-000217	JHH-6	HCC	57	Adult	Female	asian	Primary	JHH6_LIVER	1240159
ACH-000221	SNU-398	HCC	42	Adult	Male	asian	Primary	SNU398_LIVER	1240217
ACH-000316	SNU-886	HCC	57	Adult	Male	asian	Primary	SNU886_LIVER	NA
ACH-000361	SK-HEP-1	HCC	52	Adult	Male	caucasian	Metastatic	SKHEP1_LIVER	909719
ACH-000393	HLF	HCC	68	Adult	Male	asian	Primary	HLF_LIVER	NA
ACH-000420	SNU-449	HCC	52	Adult	Male	asian	Primary	SNU449_LIVER	909738
ACH-000422	SNU-475	HCC	43	Adult	Male	asian	Primary	SNU475_LIVER	909739
ACH-000471	Li-7	HCC	45	Adult	Male		Primary	LI7_LIVER	NA
ACH-000475	huH-1	HCC	53	Adult	Male	asian	Primary	HUH1_LIVER	1298146
ACH-000476	JHH-4	HCC	51	Adult	Male	asian	Primary	JHH4_LIVER	1240158
ACH-000478	SNU-387	HCC	41	Adult	Female	asian	Primary	SNU387_LIVER	909736
ACH-000480	HuH-7	HCC	57	Adult	Male	asian	Primary	HUH7_LIVER	907071
ACH-000483	SNU-182	HCC	24	Adult	Male	asian	Primary	SNU182_LIVER	1240216
ACH-000493	SNU-423	HCC	40	Adult	Male	asian	Primary	SNU423_LIVER	909737
ACH-000537	SNU-761	HCC	49	Adult	Male	asian	Primary	SNU761_LIVER	NA
ACH-000577	JHH-2	HCC	57	Adult	Male	asian	Metastatic	JHH2_LIVER	1240157
ACH-000620	JHH-1	HCC	50	Adult	Male	asian	Primary	JHH1_LIVER	1298151
ACH-000625	Hep 3B2.1-7	HCC	8	Pediatric	Male	black_or_african_american	Primary	HEP3B217_LIVER	1240147
ACH-000686	SNU-878	HCC	54	Adult	Female	asian	Primary	SNU878_LIVER	NA
ACH-000734	JHH-5	HCC	50	Adult	Male	asian	Primary	JHH5_LIVER	NA
ACH-000848	JHH-7	HCC	53	Adult	Male	asian	Primary	JHH7_LIVER	1240160
ACH-001089	HLE	HCC	68	Adult	Male	asian	Primary	HLE_LIVER	907057
ACH-001318	PLC/PRF/5	HCC	24	Adult	Male		Primary	PLCPRF5_LIVER	NA
ACH-002523	SNU-739	HCC	52	Adult	Male		Primary	SNU739_LIVER	NA
