# Supplementary material for: Application of Graph Models to the Identification of Transcriptomic Oncometabolic Pathways in Human Hepatocellular Carcinoma
Source: Biomolecules. 2024 Jun 3;14(6):653. doi: 10.3390/biom14060653 (PMC11201933; doi:10.3390/biom14060653)
Supplement: Supplementary file 1 [file biomolecules-14-00653-s001.zip › Barace_Biomolecules_SupplTable3.pdf]

Table S3. Table summary of gene set adaptation in published metabolic signatures

Signature	Core	Central	Original Gene Set	MGGC original	Size original	MGGC adapted	Size adapted
AASS	AASS, GCDH, ALDH7A1, PIPOX	AASS	REACTOME_LYSINE_CATABOLISM	0.330	12	0.549	6
ABAT	ABAT, PON3, ALDH6A1, OTC	ABAT	KEGG_ALANINE_ASPARTATE_AND_GLUTAMATE_METABOLISM<br>WP_GABA_METABOLISM_AKA_GHB<br>WP_PYRIMIDINE_METABOLISM_AND_RELATED_DISEASES	0.084	45	0.476	15
ABCC2	ABCC2, UGT2B4, UGT1A1, UGT1A9, UGT1A6	ABCC2	WP_CODEINE_AND_MORPHINE_METABOLISM	0.319	14	0.491	6
ADCY3	ADCY3, PDE10A, PDE1A, ENTPD1, PXDN	ADCY3	Purine metabolism	0.003	157	0.322	33
ADH1B	ADH1B, ADH6, ALDH2, CAT, CBR1, CYP4F11, MGST1	ADH1B	Glycolysis – Gluconeogenesis<br>KEGG_DRUG_METABOLISM_CYTOCHROME_P450<br>KEGG_RETINOL_METABOLISM<br>Miscellaneous<br>Pyruvate metabolism<br>Serotonin and melatonin biosynthesis<br>Xenobiotics metabolism	0.047	344	0.395	72
ALDH2	ABAT, ACAT1, ACSM2A, ACSM5, ADH1B, ALDH2, ALDH5A1, ALDH6A1, ALDH7A1, ALDH9A1, CAT, CYP4F2, EHHADH, GRHPR, HAGH, LDHD, MAOB, MLYCD, OTC, PCCB, PCK2, RGN	ALDH2	Ascorbate and aldarate metabolism<br>Beta-alanine metabolism<br>KEGG_ARGININE_AND_PROLINE_METABOLISM<br>KEGG_BETA_ALANINE_METABOLISM<br>KEGG_BUTANOATE_METABOLISM<br>KEGG_FATTY_ACID_METABOLISM<br>KEGG_GLYCOLYSIS_GLUCONEOGENESIS<br>KEGG_HISTIDINE_METABOLISM<br>KEGG_PROPANOATE_METABOLISM<br>KEGG_PYRUVATE_METABOLISM<br>KEGG_TRYPTOPHAN_METABOLISM<br>Leukotriene metabolism<br>Pyruvate metabolism<br>Tryptophan metabolism	0.095	177	0.464	58

ALDH6A1	ALDH6A1, MLYCD, ACADM, DBT, BCKDHB, HIBADH, MCCC2	ALDH6A1	Propanoate metabolism<br>REACTOME_BRANCHED_CHAIN_AMINO_ACID_CATABOLISM	0.163	57	0.492	18
ALOX5AP	ALOX5AP, ALOX5, TBXAS1, PTGER4, PTGS1	ALOX5AP	BIOCARTA_EICOSANOID_PATHWAY	0.256	22	0.519	9
AOX1	AOX1, MOCS2, SUOX, GPHN, MOCS1, UGT1A4, FMO3	AOX1	WP_MOLYBDENUM_COFACTOR_MOCO_BIOSYNTHESIS<br>WP_NICOTINE_METABOLISM_IN_LIVER_CELLS	0.418	12	0.539	7
B3GALT5	B3GALT5, FUT4, B3GNT7, B4GALNT4, GBGT1, GALNT7, B3GNT8	B3GALT5	Glycosphingolipid metabolism	0.015	82	0.330	18
BDH1	BDH1, ECHDC2, HADH, ACAT1, HMGCL, ECHS1	BDH1	Butanoate metabolism<br>KEGG_BUTANOATE_METABOLISM	0.149	48	0.409	14
CAT	CAT, HAO1, SCP2, CYP8B1, ABCA6, HSD17B4, AR, AQP9, ALDH9A1	CAT	HALLMARK_BILE_ACID_METABOLISM	0.155	112	0.416	34
CHST3	CHST3, CHST2, B3GNT8, B3GNT7, CHST1	CHST3	Keratan sulfate biosynthesis	0.057	23	0.407	6
COL3A1	COL3A1, COL5A1, COL1A2, COL6A3, COL6A2, ADAMTS2, COL14A1	COL3A1	REACTOME_COLLAGEN_BIOSYNTHESIS_AND_MODIFYING_ENZYMES	0.094	67	0.511	23
COX5B	COX5B, UQCRQ, UQCR11, NDUFS3, NDUFB7, COX7C, COX6B1, NDUFA2, NDUFA3, COX6A1, COX4I1, NDUFB1, NDUFA13, NDUFA4, COX5A, UQCR10, COX8A, NDUFB8, NDUFB2, COX7B, NDUFA1, NDUFS8, NDUFB10, COX6C, NDUFA8, NDUFA6, NDUFS6, NDUFA12, UQCRC1	COX5B	Oxidative phosphorylation	0.399	100	0.598	54
CPT2	CPT2, SLC25A20, CRAT, ACACB, THRSP, PRKAG2	CPT2	Carnitine shuttle (mitochondrial)<br>REACTOME_CARNITINE_METABOLISM	0.091	20	0.497	7
CYB5A	ALDH9A1, CTH, CYB5A, CYP8B1, GYS2, HSD11B1,	CYB5A	HALLMARK_GLYCOLYSIS<br>REACTOME_METABOLISM_OF_VITAMINS_AND_COFACTORS	0.037	396	0.329	107

	MTHFD1, PC, PCCB, POR, RBP4, SDC1, SOD1, SRD5A2		WP_CLASSICAL_PATHWAY_OF_STEROIDOGENESIS_WITH_GLUCOCORTICOID_AND_MINERALOCORTICOID_METABOLISM				
CYP3A4	CYP1A2, CYP2C9, CYP3A4, FMO3, UGT1A4	CYP3A4	BIOCARTA_NUCLEARRS_PATHWAY<br>REACTOME_BIOSYNTHESIS_OF_MARESIN_LIKE_SPMS<br>REACTOME_BIOSYNTHESIS_OF_MARESINS<br>REACTOME_BIOSYNTHESIS_OF_SPECIALIZED_PRORESOLVING_MEDIATORS_SPMS<br>WP_AFLATOXIN_B1_METABOLISM<br>WP_TAMOXIFEN_METABOLISM	0.156	68	0.471	15
CYP4A22	CYP4A22, CYP4A11, CYP2C8, CYP4F2	CYP4A22	WP_EICOSANOID_METABOLISM_VIA_CYTOCHROME_P450_MONOOXYGENASES_PATHWAY	0.476	9	0.516	7
CYP4F2	ACAA2, ACOX1, BAAT, CBR1, CYP2A6, CYP2C8, CYP4A11, CYP4A22, CYP4F2, CYP4F3, DHRS4, GSTK1, GSTZ1, HSD17B10, MGST1, SLC27A2	CYP4F2	Arachidonic acid metabolism<br>Drug metabolism<br>Eicosanoid metabolism<br>KEGG_ARACHIDONIC_ACID_METABOLISM	0.066	183	0.413	56
CYP8B1	ABCB11, ABCB4, ACOX2, CYP2J2, CYP4A11, CYP4A22, CYP4F2, CYP8B1, NR1I2, PON1, SCL10A1, SCP2, SLC27A5	CYP8B1	BIOCARTA_NUCLEARRS_PATHWAY<br>KEGG_PRIMARY_BILE_ACID_BIOSYNTHESIS<br>REACTOME_ARACHIDONIC_ACID_METABOLISM<br>REACTOME_BILE_ACID_AND_BILE_SALT_METABOLISM<br>WP_NUCLEAR_RECEPTORS_IN_LIPID_METABOLISM_AND_TOXICITY	0.082	128	0.476	45
DCN	DCN, BGN, VCAN, DSE	DCN	REACTOME_DERMATAN_SULFATE_BIOSYNTHESIS	0.117	11	0.497	6
DHCR7	DHCR7, EBP, LSS, NSDHL, MSMO1	DHCR7	Cholesterol biosynthesis 1 (Bloch pathway)<br>KEGG_STEROID_BIOSYNTHESIS<br>WP_CHOLESTEROL_BIOSYNTHESIS_WITH_SKELETAL_DYSPLASIAS	0.342	22	0.463	11
DHRS4	DHRS4, GSTK1, ECHS1, CBR1, MGST1, CYP4F11, HSD17B10, EPHX1	DHRS4	Arachidonic acid metabolism	0.110	111	0.369	24
DHTKD1	AASS, ALDH9A1, BCKDHB, DBT, DHTKD1, GOT2, SETD7	DHTKD1	Lysine metabolism<br>REACTOME_GLYOXYLATE_METABOLISM_AND_GLYCINE_DEGRADATION	0.052	76	0.407	18
DLD	DLD, DLST, SUCLG2P2, ACO2, SDHC	DLD	KEGG_CITRATE_CYCLE_TCA_CYCLE<br>KEGG_GLYCOLYSIS_GLUCONEOGENESIS	0.076	86	0.307	25
DMGDH	ALDH7A1, BHMT, CTH, DAO, DMGDH, MAOB, SARDH, SHMT1	DMGDH	KEGG_GLYCINE_SERINE_AND_THREONINE_METABOLISM<br>REACTOME_CHOLINE_CATABOLISM	0.319	33	0.539	14
DPYS	DPYS, XDH, UPB1, AGXT2	DPYS	REACTOME_NUCLEOTIDE_CATABOLISM	0.029	35	0.395	9

EBP	EBP, NSDHL, TM7SF2, HSD17B7	EBP	Cholesterol biosynthesis 3 (Kandustch-Russell pathway)<br>KEGG_STEROID_BIOSYNTHESIS	0.229	12	0.535	4
EHHADH	EHHADH, ACAA1, ACOX1, SLC27A5, CYP8B1, ALDH2, HSD17B4, CDO1, SCP2, ACAA2, CPT2, ETFDH, ETFA, ACOX2, CYP4F2, SLC27A2, HAO1, CAT, ABAT, ALDH6A1, SUCLG2, ALDH9A1, ECI2, MLYCD, ACBD4, HADH, HSD11B1, IVD, HIBADH, OTC, CYP4A22, CYP4A11	EHHADH	Beta oxidation of even-chain fatty acids (peroxisomal)<br>Beta oxidation of phytanic acid (peroxisomal)<br>Bile acid biosynthesis<br>Drug metabolism<br>Fatty acid oxidation<br>HALLMARK_FATTY_ACID_METABOLISM<br>KEGG_FATTY_ACID_METABOLISM<br>KEGG_PEROXISOME<br>KEGG_PROPANOATE_METABOLISM<br>Omega-3 fatty acid metabolism<br>REACTOME_FATTY_ACID_METABOLISM<br>REACTOME_PEROXISOMAL_LIPID_METABOLISM<br>Steroid metabolism<br>Valine; leucine; and isoleucine metabolism<br>WP_AMINO_ACID_METABOLISM<br>WP_EICOSANOID_METABOLISM_VIA_LIPOOXYGENASES_LOX	0.106	562	0.444	189
EP300	CREBBP, EP300, GABPA, MEF2A , SIRT1	EP300	REACTOME_REGULATION_OF_LIPID_METABOLISM_BY_PPARALPHA<br>WP_ENERGY_METABOLISM	0.037	156	0.340	40
FDPS	FDPS, MVD, HSD17B7, EBP	FDPS	REACTOME_CHOLESTEROL_BIOSYNTHESIS	0.388	27	0.514	8
FGA	FGA, FGB, FGG, MAT1A, SERPINA1	FGA	Protein assembly<br>WP_FOLATE_METABOLISM	0.105	77	0.416	24
GCDH	GCDH, ACAT1, HAAO, ALDH7A1	GCDH	KEGG_TRYPTOPHAN_METABOLISM	0.187	40	0.447	10
GLYAT	ALDH1B, ALDH2, CAT, DMGDH, GRHPR, GLYAT, GLYATL1, HAO1, HGD, HPD, TAT, UGT2B10	GLYAT	Glycine; serine and threonine metabolism<br>Phenylalanine; tyrosine and tryptophan biosynthesis	0.033	272	0.397	63
GLYATL1	GLYATL1, PIPOX, DAO, SARDH	GLYATL1	Glycine; serine and threonine metabolism	0.098	61	0.468	16
GMPPA	GMPPA, ALG3, DPM3, NANS, DPM2	GMPPA	REACTOME_BIOSYNTHESIS_OF_THE_N_GLYCAN_PRECURSOR_DOLICHOL_LIPID_LINKED_OLI GOSACCHARIDE_LLO_AND_TRANSFER_TO_A_NASCENT_PROTEIN	0.036	78	0.267	18
GNB4	GNB4, IQGAP1, GNG2, ADCY3, CACNA1C	GNB4	REACTOME_INTEGRATION_OF_ENERGY_METABOLISM	0.017	108	0.365	23

GPT	GPT, GOT2, AGXT, ASS1, CPS1, GPT2, ASL	GPT	KEGG_ALANINE_ASPARTATE_AND_GLUTAMATE_METABOLISM<br>WP_ALANINE_AND_ASPARTATE_METABOLISM	0.12	34	0.521	11
GPX4	GPX1, GPX3, GPX4, GSS, PRDX5, PRDX6	GPX4	Glutathione metabolism<br>WP_GLUTATHIONE_METABOLISM	0.051	41	0.410	8
HADH	HADH, ECHS1, ACAA2, EHHADH, ACAA1, HSD17B4, ACAT1, GSTK1, GSTZ1, MGST1, ECHDC2, ECHDC3	HADH	Fatty acid oxidation<br>Omega-6 fatty acid metabolism<br>Tryptophan metabolism<br>Vitamin E metabolism<br>WP_FATTY_ACID_BIOSYNTHESIS	0.237		0.436	50
HMGCL	HMGCL, GCDH, ACAA1, ECHS1, ECI2	HMGCL	HALLMARK_FATTY_ACID_METABOLISM	0.129	158	0.315	39
HPD	HGD, HPD, MAOB, QDPR	HPD	KEGG_TYROSINE_METABOLISM<br>REACTOME_PHENYLALANINE_AND_TYROSINE_METABOLISM	0.124	47	0.512	13
HSD17B4	ACAA1, ACSL5, AMACR, HSD17B4, MLYCD	HSD17B4	Beta oxidation of phytanic acid (peroxisomal)<br>REACTOME_PEROXISOMAL_LIPID_METABOLISM	0.308	42	0.469	15
HSD17B6	HSD17B6, SRD5A2, PIPOX, NR1I2, CES2, UGT2B10, UGT2B7, RBP4	HSD17B6	Androgen metabolism<br>HALLMARK_BILE_ACID_METABOLISM<br>Retinol metabolism<br>Steroid metabolism	0.109		0.376	68
IDI1	IDI1, HMGCR, HMGCS1, FDPS, ACAT2	IDI1	Cholesterol biosynthesis 1 (Bloch pathway)<br>Cholesterol metabolism<br>REACTOME_CHOLESTEROL_BIOSYNTHESIS<br>WP_MEVALONATE_ARM_OF_CHOLESTEROL_BIOSYNTHESIS_PATHWAY	0.157	45	0.525	22
ITPKB	ITPKB, INPP4B, INPP5D, PLCB2, PLCB4	ITPKB	REACTOME_INOSITOL_PHOSPHATE_METABOLISM	0.038	48	0.362	10
KAT2B	KAT2B, CAT, ALDH6A1, ALAD	KAT2B	HALLMARK_HEME_METABOLISM	0.025	200	0.242	52
LDHD	LDHD, HAO1, ALDH2, SDHD, ALDH9A1, GRHPR	LDHD	Tricarboxylic acid cycle and glyoxylate-dicarboxylate metabolism	0.098	49	0.479	17
LUM	LUM, PRELP, DCN, BGN, FMOD	LUM	REACTOME_DISEASES_ASSOCIATED_WITH_GLYCOSAMINOGLYCAN_METABOLISM	0.069	41	0.698	10
MAT1A	MAT1A, TAT, PON3, CTH, FMO4, CDO1, BHMT, SUOX, SAA4	MAT1A	Cysteine and methionine metabolism<br>Metabolism of other amino acids<br>REACTOME_METABOLISM_OF_INGESTED_SEMET_SEC_MESEC_INTO_H2SE<br>WP_CYSTEINE_AND_METHIONINE_CATABOLISM	0.103	126	0.430	40

			WP_METHIONINE_METABOLISM_LEADING_TO_SULFUR_AMINO_ACIDS_AND_RELATED_DISO RDERS WP_VITAMIN_B12_METABOLISM				
MCCC2	MCCC2, PCCB, PC, PCCA	MCCC2	REACTOME_DEFECTS_IN_BIOTIN_BTN_METABOLISM	0.388	8	0.456	6
NAT2	NAT2, CES1, UGT1A4, UGT1A3, CYP3A4	NAT2	KEGG_DRUG_METABOLISM_OTHER_ENZYMES	0.107	51	0.452	13
NME1	NME1, POLR2H, POLR2J, POLR2I, ITPA, NT5C, POLR2L	NME1	Nucleotide metabolism	0.009	156	0.301	36
PCK2	PCK2, SLC27A5, EHHADH, CYP8B1, SCP2, CYP4A22, ACOX2	PCK2	KEGG_PPAR_SIGNALING_PATHWAY	0.085	69	0.460	28
PIK3C2A	PIK3C2A, PIKFYVE, SYNJ1, PIK3CA, PIK3R4	PIK3C2A	KEGG_INOSITOL_PHOSPHATE_METABOLISM REACTOME_PI_METABOLISM WP_PHOSPHOINOSITIDES_METABOLISM	0.068	77	0.348	30
PIPOX	PIPOX, SARDH, GLYCTK, AGXT	PIPOX	KEGG_GLYCINE_SERINE_AND_THREONINE_METABOLISM	0.251	31	0.490	8
PSMB3	PSMB3, PSMD13, PSMC3, PSMA7, PSMC5, PSMB4, PSMC4, PSMD8	PSMB3	REACTOME_METABOLISM_OF_POLYAMINES	0.205	59	0.507	20
PXMP2	PXMP2, ECI2, PEX11G, HMGCL, GSTK1, DHRS4	PXMP2	KEGG_PEROXISOME	0.193	78	0.442	19
RPL37A	RPL37A, RPL32, RPL24, RPS14, RPL31, RPS9, RPL37, RPL29, RPL14, RPL36, RPS13, RPL27, RPL18, RPL38, RPS11, RPL19, RPL27A, RPS16, RPLP2, RPL35A, UBA52, RPL18A, RPL23, RPS8, RPL35, RPS17, RPS21, RPL28, RPS28, RPL6, RPL13A, RPL23A, RPS27A, RPL12, RPL13, RPL34, RPS23, FAU, RPS10, RPL7A, RPS7, RPS15A, RPS24, RPL11, RPL36A, RPLP1, RPL39, RPS5, RPS3	RPL37A	REACTOME_METABOLISM_OF_AMINO_ACIDS_AND_DERIVATIVES	0.051	374	0.513	115
SARDH	ASL, ASS1, CPS1, DMGDH, GOT1, HNMT, SARDH	SARDH	Sulfur metabolism WP_UREA_CYCLE_AND_METABOLISM_OF_AMINO_GROUPS	0.046	23	0.478	7

SCP2	SCP2, SAR1B, CYP8B1, SC5D, SLC27A5, HSD17B4, ABCB11, ACOX2, SLC10A1, SLC27A2, INSIG1, PPARA	SCP2	REACTOME_ALPHA_LINOLENIC_OMEGA3_AND_LINOLEIC_OMEGA6_ACID_METABOLISM<br>REACTOME_METABOLISM_OF_STEROIDS	0.070	165	0.443	43
SDHA	ASS1, CPS1, DMGDH, GOT1, HNMT, SARDH	SDHA	BIOCARTA_KREB_PATHWAY<br>REACTOME_PYRUVATE_METABOLISM_AND_CITRIC_ACID_TCA_CYCLE	0.045	33	0.478	8
SDHB	SDHB, SDHA, UQCRC1, SDHD, SLC25A4	SDHB	BIOCARTA_ETC_PATHWAY	0.303	10	0.405	7
SLC10A1	SLC10A1, SLC27A5, ABCB11, SLC27A2	SLC10A1	Bile acid recycling	0.187	16	0.443	7
SPTLC1	SPTLC1, TMPPE, ST6GALNAC3, ACER2	SPTLC1	Sphingolipid metabolism	0.016	141	0.092	29
TAT	TAT, CTH, MAT1A, HPD, PAH, MAOB, GOT2, ALDH7A1	TAT	Alanine; aspartate and glutamate metabolism<br>KEGG_CYSTEINE_AND_METHIONINE_METABOLISM<br>KEGG_PHENYLALANINE_METABOLISM<br>WP_AMINO_ACID_METABOLISM	0.061	159	0.466	37
THBS2	THBS2, LUM, ADAMTS2, DCN, PRELP, VCAN, ADAMTS12, NOTCH3, FMOD, BGN	THBS2	REACTOME_DISEASES_OF_METABOLISM	0.015	250	0.321	60
UGT1A4	UGT1A4, UGT1A3, UGT2B10, UGT2B7, MGST1, CYP1A2, CYP1A1, UGT2B4, GBE1, ALAS1, ABCG2, NAT2	UGT1A4	KEGG_ASCORBATE_AND_ALDARATE_METABOLISM<br>KEGG_METABOLISM_OF_XENOBIOTICS_BY_CYTOCHROME_P450<br>KEGG_PORPHYRIN_AND_CHLOROPHYLL_METABOLISM<br>KEGG_STARCH_AND_SUCROSE_METABOLISM<br>KEGG_STEROID_HORMONE_BIOSYNTHESIS<br>REACTOME_METABOLISM_OF_PORPHYRINS<br>WP_ARYLAMINE_METABOLISM	0.129	171	0.420	42
UGT2B10	UGT2B10, UGT2B7, CYP2C8, ADH1C, CYP3A4, CYP4A22, RDH16, CYP2A6, ADHFE1, HSD11B1, CYP2J2, SULT2A1	UGT2B10	KEGG_DRUG_METABOLISM_CYTOCHROME_P450<br>KEGG_METABOLISM_OF_XENOBIOTICS_BY_CYTOCHROME_P450<br>KEGG_RETINOL_METABOLISM<br>Linoleate metabolism<br>Xenobiotics metabolism	0.124	162	0.376	45
UGT2B7	UGT2B7, GYS2, UGT2B10, GBA3, HSD17B6, CYP3A4, HSD11B1, SRD5A2, UGT2B15, SLCO1B1, CYP2C9, CYP2C8	UGT2B7	KEGG_STARCH_AND_SUCROSE_METABOLISM<br>KEGG_STEROID_HORMONE_BIOSYNTHESIS<br>WP_CODEINE_AND_MORPHINE_METABOLISM<br>WP_TAMOXIFEN_METABOLISM	0.147	104	0.441	31

VCAN	VCAN, FMOD, LUM, XYLT1, SLC9A1, BGN, CHST3	VCAN	REACTOME_CHONDROITIN_SULFATE_BIOSYNTHESIS<br>REACTOME_CHONDROITIN_SULFATE_DERMATAN_SULFATE_METABOLISM<br>REACTOME_METABOLISM_OF_CARBOHYDRATES	0.017	246	0.371	53
